# Infantile Streptococcal Pyoderma: A Case Report

**DOI:** 10.7759/cureus.71481

**Published:** 2024-10-14

**Authors:** Masazumi Miyahara, Kyoko Osaki

**Affiliations:** 1 Department of Pediatrics, Okanami General Hospital, Iga, JPN

**Keywords:** group a streptococcus, household transmission, pharyngitis, streptococcal pyoderma, streptococcus pyogenes

## Abstract

Group A streptococcal infections in children typically present as pharyngitis but can manifest as skin infections. In infants, streptococcal skin infections can be difficult to differentiate from other skin conditions such as seborrheic or atopic dermatitis. Additionally, if another family member has a streptococcal infection or is a carrier, treating only the patient may lead to recurrence. Here, we report a case of recurrent streptococcal skin infection in a one-month-old infant with dry skin. The patient's five-year-old brother had minor nasal symptoms, and a throat swab tested positive for *Streptococcus*, identifying him as the source. The infant and his brother were both treated with antibiotics, and their conditions resolved, with no further recurrence. This case illustrates the importance of screening and treating family members in infants with streptococcal skin infections.

## Introduction

*Streptococcus pyogenes*, also known as group A *Streptococcus* (GAS), causes non-invasive human infections such as pharyngitis and pyoderma (impetigo) and can also cause severe invasive infections such as necrotizing fasciitis and streptococcal toxic shock syndrome and nonsuppurative complications such as acute glomerulonephritis and rheumatic fever. GAS skin infections can manifest as pyoderma, cellulitis, erysipelas, or necrotizing fasciitis [1]. Among them, early infantile pyoderma can develop on intertriginous areas, where skin friction and moisture create an environment conducive to bacterial growth [2], which can resemble other common conditions such as seborrheic dermatitis or atopic dermatitis, potentially leading to misdiagnosis.

Furthermore, GAS infections can be transmitted within families, where asymptomatic carriers or mildly symptomatic individuals may serve as a source of infection. Effective management of GAS skin infections in infants involves not only treating the affected infant but also identifying and addressing the source of infection within the household to prevent reinfection [3,4].

This case report aims to highlight the clinical presentation of streptococcal skin infections in young infants and emphasize the importance of screening and treating other infected family members to prevent a recurrence.

## Case presentation

**Figure 1:**
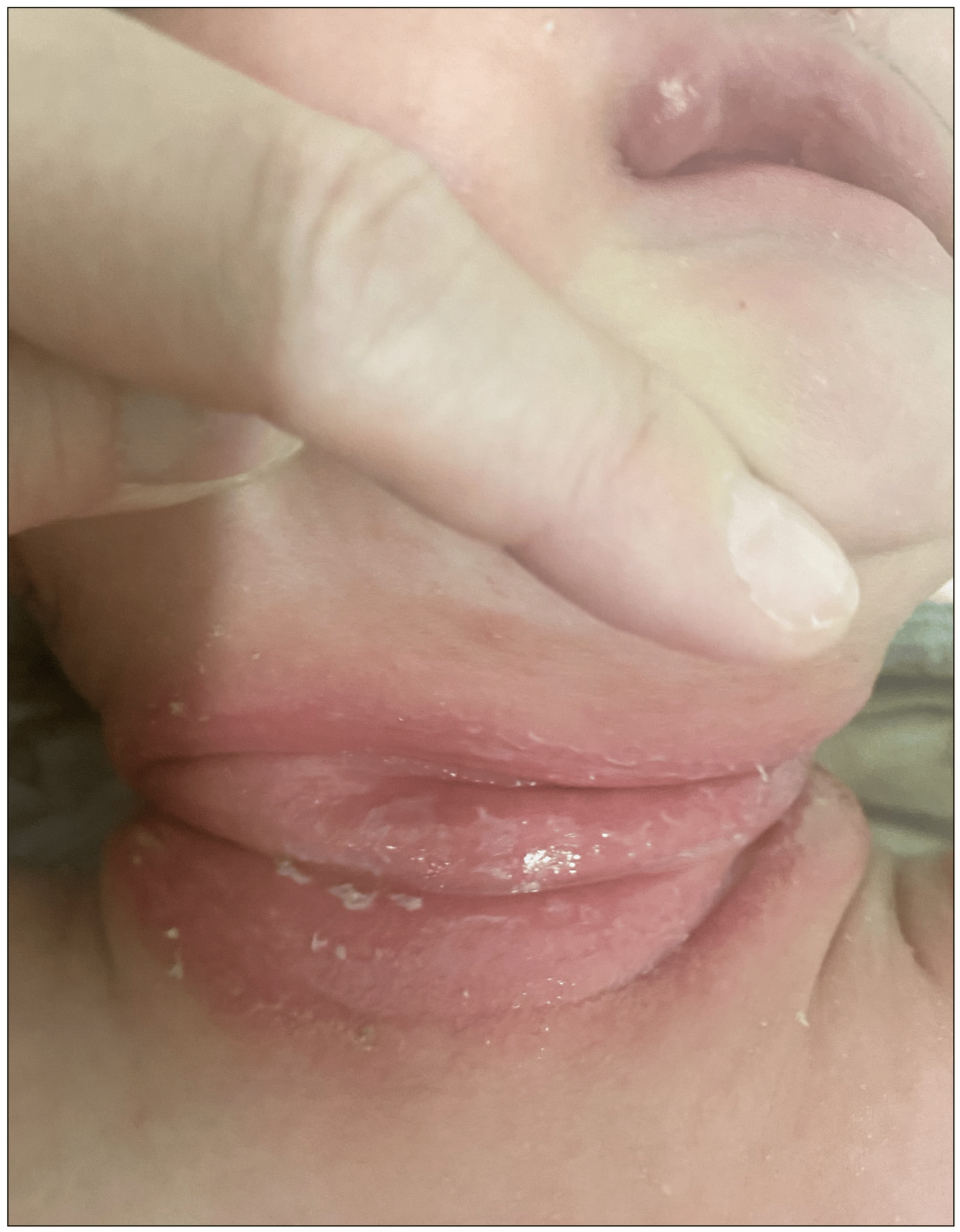
Pyoderma caused by Streptococcus pyogenes in a one-month-old infant The photograph shows a band of erythema on the skin of the neck with abrasions, exudation, and scabbing.

A previously healthy one-month-old male infant was referred to our pediatric outpatient department with a six-day history of dermatitis that had not improved after treatment with steroid cream. The infant had a temperature of 37.1℃. On examination, his neck had a band of red skin with abrasions, exudation, and scabbing (Figure 1). He had generalized dry skin, but no other physical abnormalities were noted. The patient's five-year-old brother had persistent rhinitis without fever, but no other family members had any symptoms of illness, and the patient had not had contact with anyone else with an illness. Additionally, his carers had not applied any lotions or ointments to his skin before the onset of the dermatitis. A rapid antigen test of a skin swab was positive for GAS, confirming a diagnosis of GAS pyoderma. The patient was treated with a 10-day course of oral amoxicillin and topical antibiotic cream and experienced a temporary recovery. However, three weeks later, he developed similar cutaneous lesions in the groin. Rapid GAS antigen testing of the skin lesion was again positive for GAS, and *Streptococcus pyogenes* was cultured from a skin swab of the site. The patient's brother, who had a persistent nasal discharge, was considered the likely source of infection. Therefore, when the patient's cutaneous lesions recurred, a throat swab was collected from his brother. The brother's throat swab tested positive for GAS on rapid antigen testing. Both the patient and his brother were treated with oral amoxicillin. The patient's skin lesions and his brother's rhinitis resolved and did not recur. The patient's dry skin was treated with ongoing daily application of moisturizer to maintain good skin condition and reduce the chance of a recurrence.

## Discussion

*Streptococcus pyogenes*, also known as GAS, is a group of gram-positive bacteria which commonly causes pharyngitis, pyoderma, and scarlet fever [5]. Since 2022, an increased incidence of GAS infection has been reported in multiple countries, particularly in children aged under 10 years [6]. Most attention has focused on GAS as a cause of acute pharyngitis, because of its high frequency and its potential to trigger acute rheumatic fever. However, GAS can also cause a variety of skin and soft tissue infections, some of which can lead to glomerulonephritis, and can cause severe or life-threatening disease. Although the bacterial spread in GAS skin infections is usually by direct contact and not via the respiratory tract, pyoderma serotypes can colonize the throat [7]. In this case, the bacteria most probably spread from the older brother's throat or nose to the patient's skin. In children under the age of three years, GAS pharyngitis may not manifest with fever, sore throat, or pharyngeal redness, but rather as coryza, excoriated nares, and generalized lymphadenopathy [8], making it more difficult to recognize. The patient's brother was aged five years, which is younger than the peak age group for GAS pharyngitis. He had coryza without fever, sore throat, or an erythematous pharynx, resembling the typical manifestation of GAS infection in younger children. Although the brother's coryza was nonspecific and could have been caused by allergic rhinitis or a viral upper respiratory infection, the positive result for the GAS antigen in the throat swab and the resolution of symptoms after antibiotic treatment indicate that it was caused by GAS infection. Although there is ongoing debate regarding the need for GAS screening and treatment [9,10], in cases such as this case, involving a young infant with recurrent infections, screening household members to identify hidden sources of infection is worthwhile. If infections in close contacts are overlooked and untreated, treating the patient alone, without treating the source of the infection, can lead to recurrent infections. In this case, the patient's parents were not tested to determine whether they were carriers of GAS because they were asymptomatic. The lack of recurrence of the patient's skin lesions after treating his brother with antibiotics suggests that the parents were not a source of GAS infection. Other familial risk factors include households with many residents, unsanitary living conditions, environmental tobacco smoke exposure, and low socioeconomic status. In addition, poor personal and hand hygiene and exposure to persons with asymptomatic infection are also risk factors for GAS infection [11]. The patient had close contact with his symptomatic brother, who is presumed to have a poor sense of hygiene due to his young age. Moreover, because GAS cannot penetrate intact skin, pyoderma usually occurs at the site of open lesions, such as insect bites, traumatic wounds, and burns [7]. The patient had dry skin, which is likely to have facilitated bacterial invasion. In patients with dry skin or atopic dermatitis, it is important to address these appropriately and maintain optimal skin conditions to prevent GAS infection and skin infections with other bacteria such as *Staphylococcus aureus*.

Typical GAS pyoderma manifests as numerous erythematous vesicular skin lesions, mainly on exposed areas such as the face and extremities, which rapidly progress to pustules and then rupture to form characteristic thick, honey-colored crusts, known as non-bullous impetigo [12]. However, unlike typical pyoderma, GAS skin infections in early infancy can manifest as infiltrative skin lesions with sticky secretions and are more likely to form relatively diffuse erythema in areas where the skin folds, such as the neck, inguinal region, and genital area, as in our patient. Owing to the similar appearance in the early stages, infantile pyoderma caused by GAS can be mistaken for seborrheic or atopic dermatitis. The possibility of GAS infection should be considered in all infants with eczema resistant to topical steroid treatment. Furthermore, once GAS infection is identified, it is important to take general preventive measures, such as handwashing and avoiding the sharing of items like dishes and towels, to prevent transmission within the family.

A limitation of this study is that we did not confirm that the patient and his brother were infected with the same GAS strain. M protein is a major virulence factor of GAS. Pharyngitis-associated M protein types are 1, 2, 3, 4, 5, 6, 12, 28, 75, and 89, whereas pyoderma is associated with types 33, 41, 42, 52, 53, and 70 [5]. To determine whether the infections in the patient and his brother were caused by the same strain, it would have been necessary to sequence the infecting strains or determine the type of M protein to confirm whether they matched. However, such testing is not available in general clinical practice. Given that this case occurred in early infancy and the patient had no close contact with anyone outside the family, it is likely that the infections in the patient and his brother were caused by the same strain.

## Conclusions

This case highlights the potential for GAS skin infections in infants to be misdiagnosed as other common dermatological conditions, such as seborrheic or atopic dermatitis, particularly when the lesions occur in intertriginous areas. Early diagnosis of GAS pyoderma in infants is crucial, as timely treatment with appropriate antibiotics can prevent complications and transmission to others. Typically, GAS skin infections do not occur unless there is a disruption in the skin barrier. Therefore, it is important to appropriately manage any skin abnormalities such as atopic dermatitis and dry skin, as seen in this patient.

Additionally, this case highlights the importance of identifying and treating potential sources of infection in close contacts. Although genetic testing was not conducted to confirm that the patient and his brother were infected with the same GAS strain, the clinical course strongly suggests that the GAS strains were identical. Taking preventive measures, including proper hygiene practices, is also essential to control the spread of GAS within the household.
